# Analytical Basal-State Model of the Glucose, Insulin, and C-Peptide Systems for Type 2 Diabetes

**DOI:** 10.3390/bioengineering12050553

**Published:** 2025-05-21

**Authors:** Ched C. Chichester, Munekazu Yamakuchi, Kazunori Takenouchi, Teruto Hashiguchi, Drew N. Maywar

**Affiliations:** 1 Department of Electrical and Microelectronic Engineering, Rochester Institute of Technology, Rochester, NY 14623, USA; drew.maywar@rit.edu; 2 Department of Laboratory and Vascular Medicine, Kagoshima University Graduate School of Medical and Dental Sciences, Kagoshima 890-8520, Japan; k8412148@m.kufm.kagoshimia-u.ac.jp (M.Y.); kaztak@m2.kufm.kagoshima-u.ac.jp (K.T.); k1581347@kadai.jp (T.H.); 3 Department of Electrical and Computer Engineering Technology, Rochester Institute of Technology, Rochester, NY 14623, USA

**Keywords:** mechanistic modeling, type 2 diabetes mellitus, basal state, glucose, insulin, C-peptide

## Abstract

We present a mechanistic mathematical model of the basal state for type 2 diabetes mellitus (T2DM) in an analytical form and illustrate its use for in silico basal-state and dynamic studies. At the core of the basal-state model is a quartic equation that expresses the basal plasma glucose concentration solely in terms of model parameters. This analytical model avoids a computationally intensive numerical solver and is illustrated by an investigation of how glucose-utilization parameters impact basal glucose, insulin, insulin-dependent utilization, and hepatic extraction, leveraging median parameter values of early-stage T2DM. Furthermore, the presented basal-state model ensures accurate execution of the corresponding dynamic model, which contains basal quantities within its derivative functions; erroneous, unintended dynamics in plasma glucose, insulin, and C-peptide are illustrated using an incorrect basal glucose value. The presented basal model enables efficient and accurate basal-state and dynamic studies, facilitating the understanding of T2DM pathophysiology and the development of T2DM diagnosis, treatment, and management strategies.

## 1. Introduction

Diabetes mellitus (DM) is a serious medical condition impacting hundreds of millions of people worldwide and is expected to increase in prevalence [1]. Type 2 diabetes mellitus (T2DM) is the most common form of the disease and is primarily characterized by a reduction in insulin sensitivity and, in severe cases, a reduction in insulin production [2]. T2DM is associated with many comorbidities, including hypertension, obesity, heart disease, stroke, nephropathy, retinopathy, and neuropathy [3]. The diagnosis, treatment, and management of T2DM are significantly influenced by the basal states of glucose, insulin, and C-peptides.

Basal glucose underlies the daily glucose profile. Basal glucose largely determines the fasting glucose level, which is used as a common method of diagnosing DM [4]. Moreover, basal glucose is seen as an effective means of assessing diabetes control [5]. The fasting-glucose value is also used to calculate several indices of insulin sensitivity and secretion [6]. Additionally, basal glucose acts as a “base” on top of which the postprandial glucose rises, which is another common method used to diagnose DM [4].

Endogenous basal insulin is a major component of the daily insulin profile, serving as a key indicator of insulin resistance and a predictor of impaired glucose tolerance and T2DM [7,8]. Fasting insulin is a basis of several indices of insulin sensitivity and secretion [6]. Insulin levels have been found to be a stronger metric than glucose levels for early identification of T2DM due to the tendency of insulin resistance to precede elevated glucose levels [2]. Furthermore, insulin levels are physiologically important in their own right, as their elevation is associated with many detrimental health consequences, including lipodystrophy, polycystic ovarian syndrome, and non-alcoholic fatty liver disease [9,10].

To understand the pathophysiology of DM and to assist in the development and optimization of its treatment and management, several mechanistic models of glucose and insulin dynamics have been introduced over the last 50 years [11,12,13,14,15,16,17,18]. As *mechanistic* models, these models explicitly associate parameters with physiological processes; therefore, their output is accompanied by physiological insight.

A mechanistic model for T2DM was introduced in [14] that implements a forcing-function strategy for obtaining parameter values and includes the oral glucose absorption subsystem [13], strengthening investigations into postprandial behavior such as those assessing the effectiveness and safety of insulin sensitivity testing [2]. This T2DM dynamic model has since been updated to encode subcutaneous insulin kinetics [16] and new expressions for insulin secretion and hepatic extraction [18]. The latter update is the state-of-the-art mechanistic model for T2DM and has been leveraged in recent investigations on, for example, the efficiency of smart algorithms for insulin therapy [19] and the impact of insulin therapy adherence on glucose control [20].

The dynamic model published in [18] also models C-peptide, a substance that is co-generated with insulin but with a longer half-life, negligible hepatic clearance, and a larger concentration in the plasma [21]. For these reasons, C-peptide is used to diagnose the diabetes type, detect maturity-onset diabetes of the young (MODY) [21,22], and detect latent autoimmune diabetes of adults (LADA) [21]. LADA can be distinguished from T2DM via fasting C-peptide. This fasting state, which is largely influenced by the basal state, is also used as a measure of insulin secretion and insulin resistance [6] and predicts the response to non-insulin treatments for T2DM [21].

The basal state of any dynamic physiological system is the stable steady-state solution of the corresponding dynamic model and is dependent on the model-parameter values; the value of each basal quantity cannot be set independent of the model-parameter values. The basal state of a system can be found computationally by executing, for example, a numerical solver on the model’s array of differential equations until the dynamics have damped out. Although this method allows model-parameter values to influence the basal-state solution as required, the method is indirect, computationally expensive, and prone to generate false solutions, as illustrated in this work. An *analytical* solution, if it exists, is preferred for its direct, accurate, and computationally inexpensive nature. Analytical expressions that apply to the state-of-the-art dynamic model have been reported for some basal quantities [14], but these expressions are given in terms of other basal quantities, i.e., they form a system of interconnected, *literal* equations. Analytical expressions for the basal quantities of the glucose, insulin, and C-peptide systems have not been reported solely in terms of model parameters.

An analytical, non-literal basal model corresponding to the basal state of the dynamic model reported in [18] would benefit both basal-state and dynamic studies. For basal-state studies, such a model would yield basal glucose, insulin, and C-peptide values directly, without a computationally expensive solver. Such an analytical basal-state model would also facilitate postprandial studies using the dynamic model proposed in [18], whose array of derivative functions includes basal glucose and basal insulin quantities. Accordingly, incorrect basal values, if used in the dynamic model, would be expected to produce unintended, erroneous dynamics. An analytical basal-state model would also provide correct initial conditions for studies starting with a fasted state.

The goal of the present paper is to provide an analytical basal model based solely on model parameters and to illustrate its usage for basal-state and dynamic studies. Section 2 presents the starting point for our derivation—the state-of-the-art dynamic T2DM mechanistic model published in [18] and the associated system of literal basal-state equations. Section 3 provides the derivation of the main result of the paper—the analytical basal-state model, with a quartic equation that expresses the basal plasma glucose concentration solely in terms of model parameters based on which other basal quantities can be calculated. Section 4 illustrates the application and verification of the presented basal model for both basal-state and dynamic studies, the latter of which highlights how incorrect basal values lead to erroneous dynamics. Lastly, Section 5 concludes the paper and points to future research paths.

## 2. Basal-State Model Derivation Methodology

### 2.1. Foundational Dynamic Model

**Figure 1:**
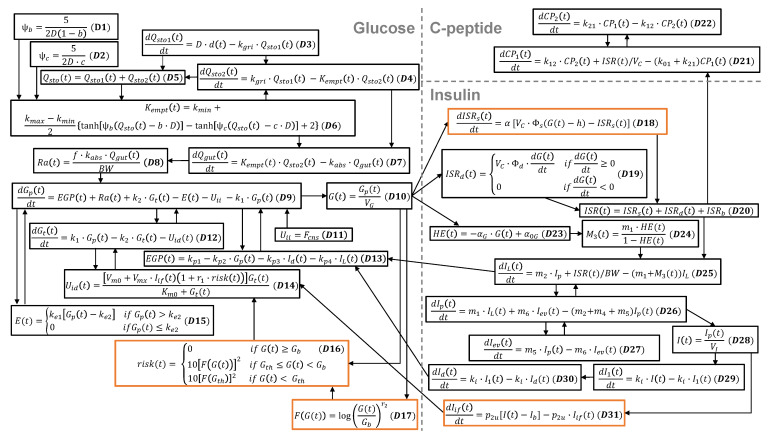
Equation schematic representation of the foundational dynamic model from [18]. The 14 differential equations and 17 algebraic equations are grouped into the glucose (left), insulin (bottom right), and C-peptide (top right) systems. Arrows explicitly show how each dynamic quantity drives the calculation of another dynamic quantity. Orange outlines highlight equations that contain the basal plasma glucose concentration ($G_{b}$, including *h*, where $h=G_{b}$) and the basal plasma insulin concentration ($I_{b}$), which are influenced by model-parameter values.

The analytical basal-state model that is the focus of our research is derived from the dynamic model reported in [18], describing the glucose, insulin, and C-peptide systems using a set of 14 differential and 17 algebraic equations representing physiological compartments, rates, and signals. To clarify the interactions between the equations within and between systems, all equations from this foundational dynamic are organized into the equation schematic representation shown in Figure 1.

This foundational dynamic model includes various parameters set to specific values, representing the influence of physiological processes that govern glucose, insulin, and C-peptide behavior. For this paper, parameter values are set to represent the median values for non-insulin-dependent, early-stage T2DM. Descriptions, units, values, and sources of all parameters are presented in Table A1.

The foundational dynamic model has an array of derivative functions that includes two basal quantities—the basal plasma glucose concentration ($G_{b}$) in Equations (D16), (D17), and (D18) where $h=G_{b}$, as well as the basal plasma insulin concentration ($I_{b}$) in Equation (D31). These basal quantities must satisfy the steady-state solution of the dynamic model. As such, they are dependent on many of the model parameters. The main objective of the work carried out in Section 3 is to show how to calculate all basal values based on model parameters in an analytical manner.

#### 2.1.1. Glucose System

The foundational dynamic model illustrated in Figure 1 contains many quantities that describe the glucose system. Oral glucose enters the glucose system at a rate of $R_{G}=D\cdot d$, where *D* (g) is the total consumed glucose dosage and *d* (1/min) is the rate of distribution over time. This distribution and our means of including it in the dynamic model are described in Appendix B.

The consumed glucose then arrives at the stomach as a total stomach glucose mass ($Q_{sto}$, mg), which is split into a solid-phase mass ($Q_{sto1}$, mg) and a liquid-phase mass ($Q_{sto2}$, mg). Glucose leaves the stomach, depending on the gastric emptying rate ($K_{empt}$, mg/min), leading to the intestine glucose mass ($Q_{gut}$, mg) and the glucose rate of appearance (Ra, mg/kg·min) [13].

The rate of appearance (Ra) drives the plasma glucose mass ($G_{p}$, mg/kg), which is directly related to the plasma glucose concentration (*G*, mg/dL). From here, there is a bidirectional transfer of glucose between $G_{p}$ and the tissue glucose mass ($G_{t}$, mg/kg); this transfer is governed by the endogenous glucose production rate (EGP, g/(kg·min)), the insulin-independent glucose utilization rate ($U_{ii}$, mg/(kg·min)), the insulin-dependent glucose utilization rate ($U_{id}$, mg/(kg·min)), and the glucose renal excretion rate (*E*, mg/(kg·min)). $U_{id}$ is further dependent on a risk function (risk) that encodes sub-basal glucose utilization [18]. As seen in Figure 1, the glucose concentration (*G*) drives multiple quantities in the insulin system.

#### 2.1.2. Insulin System

The foundational dynamic model contains many quantities that describe the insulin system. The total insulin secretion rate (ISR, pmol/min) is expressed in terms of contributions from its static $ISR_{s}$ (pmol/min), dynamic $ISR_{d}$ (pmol/min), and basal $ISR_{b}$ (pmol/min) components. The hepatic extraction ratio (HE, unitless) is assumed to be linearly dependent on *G* [23]. ISR and HE drive the liver insulin mass ($I_{L}$, pmol/kg) in counteracting ways. Insulin bidirectionally flows between $I_{L}$ and the plasma insulin mass ($I_{p}$, pmol/kg); insulin then bidirectionally flows between $I_{p}$ and the extravascular insulin mass ($I_{ev}$, pmol/kg). $I_{p}$ is directly related to the plasma insulin concentration (*I*, pmol/L), which ultimately feeds into the interstitial fluid insulin action on glucose utilization ($I_{if}$, pmol/L), the anticipated insulin action ($I_{1}$, pmol/L), and the delayed insulin action ($I_{d}$, pmol/L) [18]. As seen in Figure 1, $I_{L}$, $I_{d}$, and $I_{if}$ drive multiple quantities in the glucose system.

#### 2.1.3. C-Peptide System

The foundational dynamic model contains several quantities that describe the C-peptide system. ISR drives the C-peptide system through the accessible C-peptide concentration ($CP_{1}$, pmol/L) [18], a compartment that includes plasma and tissues that rapidly reach equilibrium with the plasma [24]. $CP_{1}$ then interacts bidirectionally with the peripheral C-peptide concentration ($CP_{2}$, pmol/L) [24], an extravascular space constituting a second C-peptide compartment.

### 2.2. System of Literal Basal-State Equations

The foundational dynamic model is leveraged to derive an analytical basal model by first setting the temporal derivatives to zero and solving for each basal quantity. Doing so yields a system of algebraic *literal* equations, that is, a system of algebraic equations whose quantities are written in terms of other quantities. Many of the equations in this system were published in [14].

Equation (D9) for the plasma glucose mass ($G_{p}$) yields its basal quantity ($G_{pb}$) [14]:

(1) $$G_{pb}=\frac{EGP_{b}+k_{2}\cdot G_{tb}-E_{b}-U_{iib}}{k_{1}},$$

where $k_{1}$ and $k_{2}$ are rate parameters and subscript *b* denotes the basal state.

For the tissue glucose concentration ($G_{t}$), Equation (D12) yields its basal quantity ($G_{tb}$):

(2) $$G_{tb}=\frac{k_{1}\cdot G_{pb}-U_{idb}}{k_{2}}.$$

For the endogenous glucose production rate (EGP), Equation (D13) readily yields its basal-state expression [14]:

(3) $$EGP_{b}=k_{p1}-k_{p2}\cdot G_{pb}-k_{p3}\cdot I_{db}-k_{p4}\cdot I_{Lb},$$

where $k_{p1}$ is the extrapolated EGP at zero glucose and insulin, $k_{p2}$ is the hepatic glucose effectiveness, $k_{p3}$ is the hepatic insulin sensitivity, and $k_{p4}$ is the portal insulin sensitivity.

The dynamic expression (D14) for the insulin-dependent glucose utilization rate ($U_{id}$) yields the basal-state equation [14]:

(4) $$U_{idb}=\frac{V_{m0}\cdot G_{tb}}{K_{m0}+G_{tb}},$$

where $V_{m0}$ and $K_{m0}$ are Michaelis–Menten kinetic parameters.

An expression for the basal glucose excretion rate ($E_{b}$) is found using dynamic Equation (D15):

(5a) $$\begin{array}{cc} E_{b} & =s\cdot k_{e1}\left(G_{pb}-k_{e2}\right), \end{array}$$

(5b) $$\begin{array}{cc} s & =\left\{\begin{array}{cc} 1 & if\;G_{pb}>k_{e2}\\ 0 & if\;G_{pb}\leq k_{e2}, \end{array}\right. \end{array}$$

where $k_{e1}$ is the glomerular filtration rate and $k_{e2}$ is the glucose renal excretion threshold. The piecewise nature of the excretion rate, as defined in [14], is moved into a multiplicative factor (*s*) to facilitate algebraic manipulation in the next section.

A basal expression for the hepatic extraction ratio (HE) is found using Equation (D23):

(6) $$HE_{b}=-\alpha_{G}G_{b}+\alpha_{0G}=-\frac{\alpha_{G}}{V_{G}}G_{pb}+\alpha_{0G},$$

where $\alpha_{G}$ is the control of glucose based on the HE, $V_{G}$ is the glucose distribution volume, and $\alpha_{0G}$ is the extrapolated HE at zero glucose. In other work, $HE_{b}$ was treated as a parameter and set to a value [18]; however, we include its expression in the system of literal basal equations to capture its role as a gateway from the glucose system to the insulin system, as shown in Figure 1.

After some algebraic manipulation, dynamic Equation (D25) for the liver insulin mass ($I_{L}$) yields the basal-state equation:

(7) $$I_{Lb}=\frac{\left(m_{2}+m_{4}\right)\left(1-HE_{b}\right)}{m_{4}+m_{2}\cdot HE_{b}}\frac{ISR_{b}}{m_{1}\cdot BW},$$

where $m_{1}$, $m_{2}$, and $m_{4}$ are rate parameters and BW is body weight. A basal-state expression for the delayed insulin ($I_{d}$) is derived from Equation (D30) as

(8) $$I_{db}=I_{Lb}\left[\frac{m_{1}}{V_{I}\left(m_{2}+m_{4}\right)}\right],$$

where $V_{I}$ is the insulin distribution volume.

The remaining eight dynamic quantities are nullified in the steady state. The rate of appearance ($Ra_{b}$) and all digestive compartments ($Q_{sto1b}$, $Q_{sto2b}$, $Q_{stob}$, and $Q_{gutb}$) equal zero because the consumed glucose dosage is D=0. The insulin secretion rates ($ISR_{sb}$ and $ISR_{db}$) equal zero because, in a basal state, $G=G_{b}$ and dG/dt=0. The interstitial-fluid insulin action is $I_{ifb}=0$ because $I=I_{b}$ in a basal state.

### 2.3. Methodology for Solving the System of Literal Basal-State Equations

The system of Equations (1)–(8) expresses basal quantities in terms of other basal quantities. This system of literal basal equations can be “unwrapped” using algebra such that each quantity or, more simply, a single core quantity is written solely in terms of model parameters, without any other unknown basal quantity. For this effort, we select the basal plasma glucose concentration ($G_{b}$) as this core quantity. Doing so yields a *quartic* equation for $G_{b}$, which can be solved analytically. The multiple mathematical solutions of the quartic equation are examined using parameter values published in the literature. The following section details this derivation and the key result of our paper: the analytical basal model.

## 3. Analytical Basal-State Model—Derivation and Result

### 3.1. Quartic Equation for $G_{b}$

An expression for the basal-glucose concentration ($G_{b}$) solely in terms of model parameters can be derived from the system of literal basal-state Equations ((1)–(8)). Equations (3), (7), and (8) combine to yield

(9a) $$\begin{array}{cc} EGP_{b} & =k_{p1}-k_{p2}\cdot G_{pb}-\varepsilon \frac{\left(m_{2}+m_{4}\right)\left(1-HE_{b}\right)}{m_{4}+m_{2}\cdot HE_{b}}, \end{array}$$

(9b) $$\begin{array}{cc} \varepsilon & =\left(\frac{k_{p3}\cdot m_{1}}{V_{I}\left(m_{2}+m_{4}\right)}+k_{p4}\right)\frac{ISR_{b}}{m_{1}\cdot BW}. \end{array}$$

Equation (6) is then substituted into Equation (9a) to yield

(10a) $$\begin{array}{c} EGP_{b}=k_{p1}-k_{p2}\cdot G_{pb}-\frac{\zeta +\eta \cdot G_{pb}}{\theta +\lambda \cdot G_{pb}}, \end{array}$$

(10b) $$\begin{array}{cc} \zeta =\varepsilon \left(m_{2}+m_{4}-m_{2}\cdot \alpha_{0G}-m_{4}\cdot \alpha_{0G}\right), & \theta =m_{4}+m_{2}\cdot \alpha_{0G}, \end{array}$$

(10c) $$\begin{array}{cc} \eta =\varepsilon \cdot \alpha_{G}\left(m_{2}+m_{4}\right)/V_{G}, & \lambda =-m_{2}\cdot \alpha_{G}/V_{G}. \end{array}$$

Equations (3) and (5a) are then substituted into Equation (1) to, after manipulation, yield

(11a) $$\begin{array}{c} k_{2}\cdot G_{tb}=\mu G_{pb}+\frac{\zeta +\eta G_{pb}}{\theta +\lambda G_{pb}}-\rho , \end{array}$$

(11b) $$\begin{array}{cc} \mu =k_{1}+k_{p2}+s\cdot k_{e1}, & \rho =k_{p1}+s\cdot k_{e1}\cdot k_{e2}-U_{iib}. \end{array}$$

A second expression for $G_{tb}$ is found by substituting Equation (4) into Equation (2):

(12) $$k_{2}\cdot G_{tb}=k_{1}\cdot G_{pb}-\frac{V_{m0}\cdot G_{tb}}{K_{m0}+G_{tb}}.$$

Equations (11a) and (12) are a system of two equations for the two unknowns ($G_{pb}$ and $G_{tb}$). These equations can be combined to yield an expression for $G_{pb}$ only in terms of model parameters:

(13) $$\left(k_{1}-\mu \right)G_{pb}-\frac{\frac{V_{m0}}{k_{2}}\left(\mu G_{pb}+\frac{\zeta +\eta G_{pb}}{\theta +\lambda G_{pb}}-\rho \right)}{K_{m0}+\frac{1}{k_{2}}\left(\mu G_{pb}+\frac{\zeta +\eta G_{pb}}{\theta +\lambda G_{pb}}-\rho \right)}-\frac{\zeta +\eta G_{pb}}{\theta +\lambda G_{pb}}+\rho =0.$$

The basal plasma glucose mass ($G_{pb}$) is related to the basal plasma glucose concentration ($G_{b}$) by

(14) $$G_{pb}=V_{G}G_{b},$$

the basal form of Equation (D10). Thus, Equation (13) can be rewritten in terms of $G_{b}$ as

(15) $$\left(k_{1}-\mu \right)V_{G}G_{b}-\frac{\frac{V_{m0}}{k_{2}}\left(\mu V_{G}G_{b}+\frac{\zeta +\eta V_{G}G_{b}}{\theta +\lambda V_{G}G_{b}}-\rho \right)}{K_{m0}+\frac{1}{k_{2}}\left(\mu V_{G}G_{b}+\frac{\zeta +\eta V_{G}G_{b}}{\theta +\lambda V_{G}G_{b}}-\rho \right)}-\frac{\zeta +\eta V_{G}G_{b}}{\theta +\lambda V_{G}G_{b}}+\rho =0.$$

In principle, Equation (15) can be used to calculate $G_{b}$ via a computational root-finding algorithm. However, many such numerical algorithms fail to find multiple roots over a defined interval of $G_{b}$. Furthermore, such algorithms can yield false roots when a denominator in the expression under study passes through zero.

Alternatively, a numerical root-finding algorithm can be avoided by developing a closed-form analytical solution for $G_{b}$. We do so by algebraically converting Equation (15) into the following quartic polynomial expression:

(16) $$aG_{b}^{4}+bG_{b}^{3}+cG_{b}^{2}+dG_{b}+e=0,$$

where

(17a) $$\begin{array}{cc} a/V_{G}^{4} & =\mu \lambda^{2}\left(k_{1}-\mu \right), \end{array}$$

(17b) $$\begin{array}{ccc} b/V_{G}^{3} & = & \mu \lambda (k_{1}\theta -\mu \theta +\rho \lambda -\eta )\\ & & +\lambda \left(k_{1}-\mu \right)\left(\mu \theta -\rho \lambda +\eta +k_{2}K_{m0}\lambda \right)-V_{m0}\mu \lambda^{2}, \end{array}$$

(17c) $$\begin{array}{ccc} c/V_{G}^{2} & = & \left(\rho \theta -\zeta \right)\mu \lambda +\left(\mu \theta -\rho \lambda +\eta +k_{2}K_{m0}\lambda \right)\left(k_{1}\theta -\mu \theta +\rho \lambda -\eta \right)\\ & & +\lambda \left(k_{2}K_{m0}\theta -\rho \theta +\zeta \right)\left(k_{1}-\mu \right)-V_{m0}\lambda \left(2\theta \mu +\eta -\rho \lambda \right), \end{array}$$

(17d) $$\begin{array}{ccc} d/V_{G} & = & \left(\rho \theta -\zeta \right)\left(\mu \theta -\rho \lambda +\eta +k_{2}K_{m0}\lambda \right)+\left(k_{2}K_{m0}\theta -\rho \theta +\zeta \right)\left(k_{1}\theta -\mu \theta +\rho \lambda -\eta \right)\\ & & -V_{m0}\left(\mu \theta^{2}+\theta \eta +\lambda \zeta -2\lambda \rho \theta \right), \end{array}$$

(17e) $$\begin{array}{cc} e & =\begin{array}{c} \left(k_{2}K_{m0}\theta -\rho \theta +\zeta \right)\left(\rho \theta -\zeta \right)-V_{m0}\theta \left(\zeta -\rho \theta \right). \end{array} \end{array}$$

This quartic expression can be solved with Ferrari’s method to yield

(18) $$G_{bk\pm_{1}\pm_{2}}=-\frac{b}{4a}+\frac{\pm_{1}\sqrt{2m_{k}}\pm_{2}\sqrt{-\left(2p+2m_{k}\pm_{1}q\sqrt{\frac{2}{m_{k}}}\;\right)}}{2},$$

where *p* and *q* are coefficients of the associated depressed-quartic equation:

(19) $$p=\frac{8ac-3b^{2}}{8a^{2}},\;\;\;\;\;\;\;\;\;\;\;\;q=\frac{b^{3}-4abc+8a^{2}d}{8a^{3}},$$

and $m_{k}$ is a root of the associated resolvent cubic equation, where k∈{1,2,3}. The root ($m_{k}$) is found using Cardano’s method:

(20a) $$\begin{array}{c} m_{k}=-\frac{1}{24}\left(8p+\xi^{k-1}\varepsilon +\frac{\Delta_{0}}{\varepsilon }\right), \end{array}$$

(20b) $$\begin{array}{cc} \xi =\frac{-1+\sqrt{-3}}{2}, & \varepsilon =\sqrt[{3}]{\frac{\Delta_{1}+\sqrt{\Delta_{1}^{2}-4\Delta_{0}^{3}}}{2}}, \end{array}$$

(20c) $$\begin{array}{cc} \Delta_{0}=\frac{16(c^{2}-3bd+12ae)}{a^{2}}, & \Delta_{1}=\frac{64(-2c^{3}-27\left(b^{2}e+ad^{2}\right)+9bcd+72ace)}{a^{3}}, \end{array}$$

where ξ is a primitive cube root of unity.

### 3.2. The Full Basal-State Model

**Figure 2:**
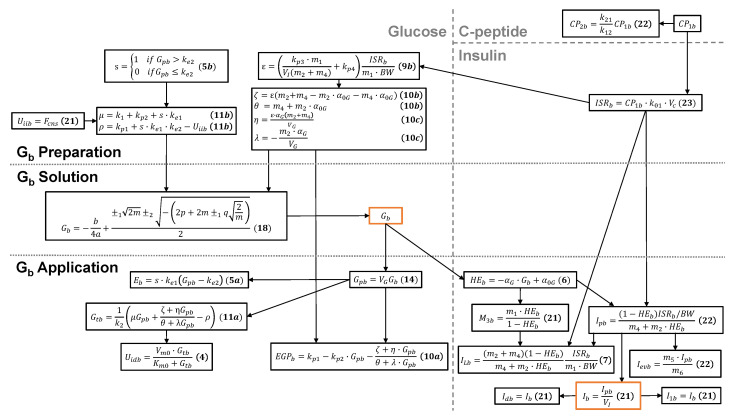
Equation schematic representation and process flow of the derived analytical basal-state model. The $G_{b}$ *preparation* layer calculates a range of quantities in preparation for Equation (18) for $G_{b}$. The $G_{b}$ *solution* layer solves the analytical quartic equation for $G_{b}$ based solely on model-parameter values. The calculated $G_{b}$ then flows into the $G_{b}$ *application* layer, wherein the remaining basal values are calculated. The physical value of $G_{b}$ is determined as discussed in Section 3.3. $G_{b}$ and $I_{b}$, which appear in the derivative functions of the foundational dynamic model, are outlined in orange.

The basal glucose ($G_{b}$) and the full basal-state model are depicted in Figure 2. The $G_{b}$ *preparation* layer is executed to calculate the parameters needed for Equation (18), which is calculated in the $G_{b}$ *solution* layer. Since $G_{b}$ is defined by model parameters, it serves as the core quantity through which to express the remaining basal quantities.

Once $G_{b}$ is found, the $G_{b}$ *application* layer is executed to calculate the remaining basal values directly using the equations reported in the figure. Several of these remaining basal quantities do not appear in the system of literal basal-state equations and are reported in [14]:

(21) $$U_{iib}=F_{cns},\;\;\;\;\;\;\;\;\;\;M_{3b}=\frac{m_{1}\cdot HE_{b}}{1-HE_{b}},\;\;\;\;\;\;\;\;\;\;I_{b}=I_{pb}/V_{I},\;\;\;\;\;\;\;\;\;\;I_{1b}=I_{b},\;\;\;\;\;\;\;\;\;\;I_{db}=I_{b},$$

as well as in [18]:

(22) $$CP_{2b}=\frac{k_{21}}{k_{12}}CP_{1b},\;\;\;\;\;\;\;\;\;\;\;\;I_{pb}=\frac{\left(1-HE_{b}\right)ISR_{b}/BW}{m_{4}+m_{2}\cdot HE_{b}},\;\;\;\;\;\;\;\;\;\;\;\;I_{evb}=\frac{m_{5}\cdot I_{pb}}{m_{6}},$$

(23) $$ISR_{b}=CP_{1b}\cdot k_{01}\cdot V_{c}.$$

Note that Equation (23) expresses the basal rate of insulin secretion ($ISR_{b}$) in terms of the basally accessible C-peptide concentration ($CP_{1b}$). This simple relationship between $ISR_{b}$ and $CP_{1b}$ allows either quantity to be calculated based on the other. A benefit of expressing $ISR_{b}$ in terms of $CP_{1b}$ is that the latter is more directly measurable and, thus, carries less uncertainty; for this reason, the concentration of $CP_{1b}$ appears in Table A1.

### 3.3. Identifying Physical Basal Solutions

The quantity of $G_{bk\pm_{1}\pm_{2}}$, as expressed by Equation (18), has 12 mathematical solutions, spanning the scope of *k*, $\pm_{1}$, and $\pm_{2}$. Checking the physical nature of these solutions must be a part of executing the basal model. To this end, all values of *k*, $\pm_{1}$, and $\pm_{2}$ are shown in Table 1 for the median early-stage T2DM parameter values presented in Table A1.

Examining the k=1 case reveals that several calculated basal quantities are negative and, therefore, nonphysical and should be discarded from consideration. For the “++” and “−+” configurations, $HE_{b}<0$ and $I_{b}<0$; all such nonphysical values are reported in the table using red font. For the “−−” configuration, $G_{b}<0$, $I_{b}<0$, and $HE_{b}>1$. For the “+−” configuration, no basal quantity is identified as nonphysical; therefore, this solution is interpreted as physically correct. Note that for the parameter values used in this example, $HE_{b}$ and $I_{b}$ each independently isolates the single physical solution, whereas $G_{b}$ and $EGP_{b}$ do not.

Examining the k={2,3} cases reveals the same basal values found for the k=1 case but for different “$\pm_{1}\pm_{2}$” configurations. In particular, the physically correct solution occurs for the “−+” and “+−” configurations for k=2 and 3, respectively. Thus, each *k* value yields the same physically correct solution and can be used. For the remainder of this paper, we use $k=2,\pm_{1}=-$, and $\pm_{2}=+$.

## 4. Analytical Basal-State Model—Applications and Model Verification

### 4.1. Basal Values Corresponding to Reported Median T2DM Parameter Values

The median early-stage T2DM model-parameter values presented in Table A1 are reported for the foundational dynamic model; however, not all corresponding basal values have been previously reported. The basal values are calculated here via the presented basal-state model and are listed in Table 2. The calculated values of $G_{b}$ and $I_{b}$ appear to match the initial values in plots of modeled dynamic profiles published in [18]; other calculated basal quantities do not have such a reference. The calculated values of $G_{b}$ and $I_{b}$ also appear to match the initial values in plots of measured dynamic profiles published in [18].

### 4.2. Basal Model Parameter Study

**Figure 3:**
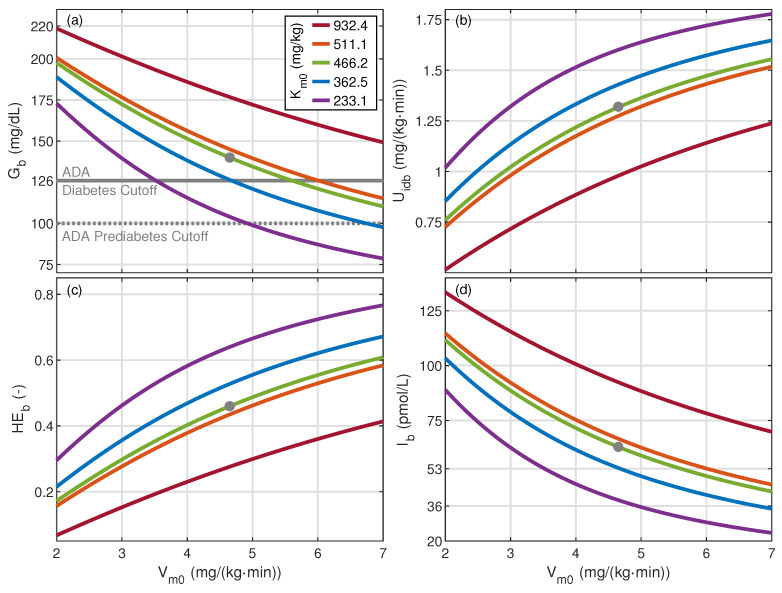
Impact of the glucose utilization parameters $V_{m0}$ and $K_{m0}$ on basal (**a**) glucose ($G_{b}$), (**b**) insulin-dependent utilization ($U_{idb}$), (**c**) hepatic extraction of insulin ($HE_{b}$), and (**d**) insulin ($I_{b}$), leveraging the process flow shown in Figure 2. The resulting $G_{b}$ and $I_{b}$ values decrease with an increase in $V_{m0}$ and a decrease in $K_{m0}$, whereas $U_{idb}$ and $HE_{b}$ increase under these conditions. The ADA fasting-based glucose cutoffs for diagnosis of diabetes (126 mg/dL) and pre-diabetes (100 mg/dL) are shown in (**a**). Each basal value using the median early-stage T2DM case shown in Table A1 is denoted by a solid gray circle.

The presented analytical basal-state model permits the direct execution of parameter studies, whereby variation in parameter values provides insight into the basal-state pathophysiology of T2DM. As an illustration, we execute a parameter study using the Michaelis–Menten parameters ($V_{m0}$ and $K_{m0}$) of the insulin-dependent glucose utilization ($U_{idb}$). According to Equation (4), the $V_{m0}$ parameter is the maximum value of $U_{idb}$, and the $K_{m0}$ parameter is the value of $G_{tb}$ for which $U_{idb}=V_{m0}/2$. For this study, the value of $K_{m0}$ presented in Table A1 is augmented to $K_{m0}=\left\{233.1,362.5,466.2,511.1,932.4\right\}$ mg/kg, which are half the median, the 25th percentile, the median, the 75th percentile, and double the median, respectively, of the percentile data reported in [18]. Furthermore, the value of $V_{m0}$ presented in Table A1 is augmented to a range of 2.00 to 7.00 mg/(kg·min). The impact of $K_{m0}$ and $V_{m0}$ on each basal quantity is significant, as illustrated in Figure 3 for $G_{b}$, $HE_{b}$, $U_{idb}$, and $I_{b}$—quantities chosen to express T2DM pathophysiology.

The calculated $G_{b}$ increases as $V_{m0}$ decreases and as $K_{m0}$ increases, as shown in Figure 3a; quantitatively, $G_{b}$ varies from 78.65 to 218.49 mg/dL. This significant variation intersects the cutoffs used for diabetes diagnosis based on fasting plasma glucose. For example, the American Diabetes Association (ADA) classifies diabetes as 126 mg/dL and above, normal as less than 100 mg/dL, and pre-diabetes as the intermediate region starting at 100 mg/dL; these cutoffs are highlighted in Figure 3a.

The insulin-dependent glucose utilization rate ($U_{idb}$) decreases as $V_{m0}$ decreases and as $K_{m0}$ increases, as expected by Equation (4), whose parameters are varied in this study. Quantitatively, $U_{idb}$ varies from 0.52 to 1.78 mg/(kg·min). A decrease in this utilization rate slows the removal of glucose by the tissues, ultimately increasing $G_{b}$, as seen in Figure 3a.

The basal hepatic extraction ratio of insulin ($HE_{b}$) also decreases as $V_{m0}$ decreases and as $K_{m0}$ increases, as shown in Figure 3c, with a range of 0.07 to 0.77. Lower $HE_{b}$ is associated with higher $G_{b}$, as expected from Equation (6). The physiological mechanisms of $U_{idb}$ and $HE_{b}$ are not directly accessible through standard lab tests; parameter studies such as this can provide insight into their behavior and association with measurable quantities like $G_{b}$ and $I_{b}$.

Basal insulin ($I_{b}$) increases as $V_{m0}$ decreases and as $K_{m0}$ increases, as seen in Figure 3d, with a total variation of 23.73 to 133.44 pmol/L. Higher basal insulin is associated with a lower rate of hepatic extraction ($HE_{b}$), as expected from Equations (D24) and (D25). Although a lower $HE_{b}$ promotes the concentration of insulin, lower $HE_{b}$ is evidently accompanied by lower insulin-dependent utilization ($U_{idb}$). The net effect is that higher basal insulin is associated with higher basal glucose, a common occurrence for T2DM.

### 4.3. Model Verification

**Figure 4:**
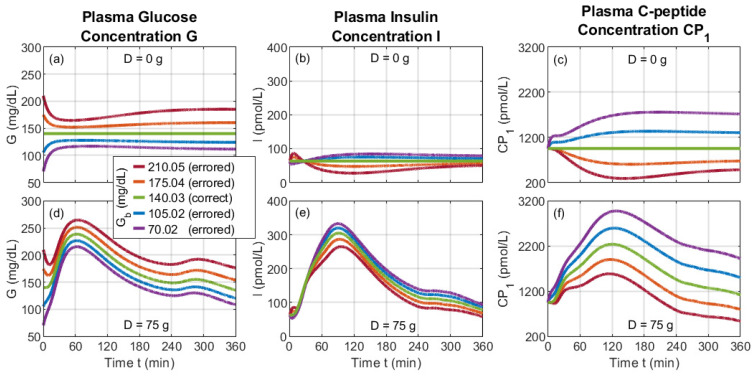
Impact of incorrect $G_{b}$ values on the dynamic temporal profiles of glucose, insulin, and C-peptide for oral glucose doses of (**a**–**c**) D=0 g and (**d**–**f**) D=75 g. For D=0 g, which characterizes the top row, the correct basal value results in a flat temporal profile (green lines), whereas the incorrect basal values result in physically incorrect dips and ledge-like variations as well as easy-to-misinterpret, steady-state-like levels. For D=75 g, which characterizes the bottom row, incorrect basal values produce incorrect preprandial and postprandial dynamics.

One method to verify an analytical basal-state model is to use its basal-value outputs as initial conditions applied to its corresponding dynamic model for the case of a *zero* consumed glucose dose (*D*). Under these conditions, the dynamic profile of each quantity should remain flat over time, at a value equal to its initial condition. To this end, we coded the foundational dynamic model in MATLAB (version 24.2) and solved it numerically using an ODE solver, the details of which are presented in Appendix B. Parameter values are presented in Table A1. Under the D=0 g condition, the modeled quantities, indeed, remained fixed, as represented by the *green* curves of Figure 4a for the plasma glucose concentration (*G*), Figure 4b for the plasma insulin concentration (*I*), and Figure 4c for the plasma C-peptide concentration ($CP_{1}$).

Well-behaved basal-state solutions for parametric studies like that reported in Section 4.2 should use “$\pm_{1}\pm_{2}$” configurations that maintain physically correct solutions over the entire range of parameter values. Using k=1 for the parameter study discussed in Section 4.2 is an example for which the “$\pm_{1}\pm_{2}$” configuration needs to change to maintain physically correct solutions; this change occurs when $m_{k}$ approaches zero. Using k={2,3} does not require a change in the “$\pm_{1}\pm_{2}$” configuration.

Viable basal-state solutions are also stable against perturbations. We test this stability numerically using the foundational dynamic model and a perturbation of an oral glucose dose of D=75 g. Under this condition, the modeled quantities, indeed, return to the basal values after postprandial dynamics. This behavior is represented by the *green* curves of Figure 4d for the plasma glucose concentration (*G*), Figure 4e for the plasma insulin concentration (*I*), and Figure 4f for the plasma C-peptide concentration ($CP_{1}$).

### 4.4. Misusing the Dynamic Model via Incorrect Basal Values

The foundational dynamic model requires basal-state values as part of its system of derivative functions, as depicted in Figure 1, and is often executed with initial conditions set to basal-state values. As such, errors in the basal-state values are expected to erroneously impact the output of the dynamic model. As an example, we introduce an erroneous basal value for $G_{b}$, a quantity found in the derivative function for $ISR_{s}$ Equation (D18) via *h* and in the risk function (Equations (D16) and (D17)), as shown in Figure 1. For the median early-stage T2DM case, whose parameter values are presented in Table A1, the correct $G_{b}$ is calculated by the basal-state model to be 140.03 mg/dL. For this study, $G_{b}$ = {70.02, 105.02, 140.03, 175.04, 210.05} mg/dL, where the erroneous values vary from 50% below to 50% above the associated correct basal value.

Without oral glucose (D=0 g), the correct $G_{b}$ value results in a steady value for *G*, *I*, and $CP_{1}$, as shown in Figure 4a–c. However, an incorrect $G_{b}$ value results in various incorrect short-term and long-term behavior. In the early timescale of t<60 min, significant changes occur in *G*, *I*, and $CP_{1}$. Erroneously small values of $G_{b}$ yield a ledge-like feature in $CP_{1}$. At longer time scales, *G*, *I*, and $CP_{1}$ each appear to settle at a flat, steady-state-like value that is different than the initial condition and, importantly, different than the correct basal value; this difference is driven by the appearance of $G_{b}$ in the derivative function for $ISR_{s}$. Thus, this numerical method for determining the basal state of the system (i.e., by running a differential equation solver until dynamics damp out) is prone to misinterpretation, further motivating the analytical basal-state model presented in this work.

For the case *with* oral glucose, the impact of the incorrect $G_{b}$ values is readily apparent in the temporal profiles of *G*, *I*, and $CP_{1}$, as seen in Figure 4d–f for an oral glucose dose of D=75 g. This dosage and associated rate of distribution (d(t)) are consistent with an oral glucose tolerance test (OGTT) standard [25], as defined in Appendix B. In the early timescale of t<15 min, profiles vary similarly to the case without oral glucose, then dovetail into a surge due to oral glucose. This behavior leads to a dip in *G* for erroneously high $G_{b}$. The splay between *G* curves narrows near t=120 min, then opens at longer times. The *I* and $CP_{1}$ profiles begin at the same $I_{b}$ and $CP_{1b}$ values for all $G_{b}$ cases. At longer timescales, *I* and $CP_{1}$ profiles are splayed by an amount relatively smaller and larger than that of *G*, respectively. For each quantity, the widest variation over the profile occurs for $G_{b}=70.02$ mg/dL. Conversely, the smallest variation occurs for $G_{b}=210.05$ mg/dL, albeit with prominent redirection at early timescales.

## 5. Discussion

The main result of this paper is the presentation of an analytical, mechanistic model that allows the basal state of the glucose, insulin, and C-peptide systems to be calculated directly based on the model-parameter values. This model, derived from the mechanistic dynamic model presented in [18], has, at its core, a quartic equation for basal glucose ($G_{b}$) expressed solely in terms of model parameters. Fortuitously, a polynomial equation that is of quartic or lower order has an analytical solution, whereas that of a quintic or higher order does not. The solution for $G_{b}$ is then applied to calculate the remaining basal quantities.

The quartic nature of the basal glucose $G_{bk\pm_{1}\pm_{2}}$ expression yields four roots ($\pm_{1}$, $\pm_{2}$) for each of the three roots ($m_{k}$) of the associated resolvent cubic equation. We find that for the median early-stage T2DM parameter values, only one basal state is physical for each $m_{k}$ value. However, there may exist sets of parameter values that yield *multiple* physical basal states. Future research may uncover such sets, which may lead to further model development to investigate potentially new and interesting behavior.

The presented basal-state model facilitates correct basal-state and dynamic studies. For basal-state studies, the presented model provides solutions directly; an alternative method of executing a differential equation solver until the dynamics damp out can yield steady-state-like values that are easy to misinterpret as basal values, as seen in the top row of Figure 4. For dynamic studies, the presented basal-state model avoids erroneous dynamic profiles resulting from incorrect basal values; this benefit is seen in the bottom row of Figure 4 using erroneousvalues of the basal plasma glucose ($G_{b}$) and also occurs for other basal quantities, whether or not they appear in a derivative function.

The scope of biological substances and processes of the presented basal model is explicitly shown in Figure 2. The substances therein are listed concisely in Table 2, plus the accessible C-Peptide concentration listed in Table A1; substances not found here, such as glucagon, incretins, and exogenous insulin, are not included in the basal model. Future model development efforts could incorporate such additional substances to expand the model’s application. However, some additional substances might not occur in the basal state; glucagon is included in the T1DM proposed in [17], where it is clear that glucagon does not impact the basal state. (Note that [17] shows that glucagon only impacts the postprandial state at time intervals t > 360 min; accordingly, we restrict the in silico dynamic results in Section 4 to t ≤ 360 min.) The processes of the basal model are listed concisely in Table A1; processes not represented here, such as time-varying parameter values, are not included in the basal model. Future model development efforts could incorporate such additional processes; however, some additional processes might be interpretable within the current model. For example, insulin sensitivity is known to vary diurnally [26,27], which can impact basal values. Future work can include such time dependence in the current basal-state model, with the assumption that the rates of change expressed by the dynamic model are much faster than the diurnal variation in parameter values, such that the calculated basal state adiabatically follows the relatively slow time variation in the model-parameter values.

The pathophysiology of T2DM and the development of strategies for its diagnosis, treatment, and management can be studied by varying targeted model parameters. Whereas the parameter values investigated in the present paper are based on median early-stage T2DM, other populations and distributions could also be insightful. For example, parameters values representing pre-diabetic and non-diabetic subjects can be explored. Moreover, future studies can apply statistical methods such as those presented in [17] to generate random populations of virtual patients with inter-subject variability. Such sets of parameter values can be applied to the presented basal-state model to assist in the understanding, diagnosis, treatment, and management of T2DM.

## Figures and Tables

**Table 1:** The basal-state model yields 12 mathematical solutions for each basal quantity, as determined by *k*, $\pm_{1}$, and $\pm_{2}$. Physically correct values are found by disregarding cases for which any quantity is negative or $HE_{b}>1$ (shown in red).

*k*	$\pm_{1}$	$\pm_{2}$	$G_{b}$ (mg/dL)	${\mathrm{EGP}}_{b}$ (mg/(kg·min))	${\mathrm{HE}}_{b}$ (unitless)	$I_{b}$ (pmol/L)
1	+	+	586.92	61.58	−1.77	−5618.51
1	+	−	140.03	2.32	0.46	62.95
1	−	+	1669.25	5.58	−7.19	−364.28
1	−	−	−1806.42	6.54	10.19	−191.08
2	+	+	1669.25	5.58	−7.19	−364.28
2	+	−	586.92	61.58	−1.77	−5618.51
2	−	+	140.03	2.32	0.46	62.95
2	−	−	−1806.42	6.54	10.19	−191.08
3	+	+	1669.25	5.58	−7.19	−364.28
3	+	−	140.03	2.32	0.46	62.95
3	−	+	586.92	61.58	−1.77	−5618.51
3	−	−	−1806.42	6.54	10.19	−191.08

**Table 2:** The full set of basal values calculated using the presented basal-state model with the median early-stage T2DM parameter values presented in Table A1. Basal quantities are grouped by their presence in the glucose, insulin, and C-peptide systems.

System	Symbol	Basal Quantity Description	Unit	Value
Glucose	$G_{b}$	Plasma-glucose concentration	mg/dL	140.03
	$G_{pb}$	Plasma-glucose mass	mg/kg	140.03
	$EGP_{b}$	Endogenous glucose-production rate	mg/(kg·min)	2.32
	$E_{b}$	Glucose-excretion rate	mg/(kg·min)	0
	$G_{tb}$	Tissue-glucose mass	mg/kg	184.30
	$U_{idb}$	Insulin-dependent utilization rate	mg/(kg·min)	1.32
	$U_{iib}$	Insulin-independent utilization rate	mg/(kg·min)	1
Insulin	$ISR_{b}$	Insulin-secretion rate	pmol/min	246.20
	$HE_{b}$	Hepatic-extraction ratio	unitless	0.46
	$M_{3b}$	Hepatic-extraction parameter	1/min	0.27
	$I_{pb}$	Plasma-insulin mass	pmol/kg	2.58
	$I_{Lb}$	Liver-insulin mass	pmol/kg	5.84
	$I_{evb}$	Extravascular-insulin mass	pmol/kg	39.47
	$I_{b}$	Plasma-insulin concentration	pmol/L	62.95
	$I_{1b}$	Anticipated insulin signal	pmol/L	62.95
	$I_{db}$	Delayed insulin signal	pmol/L	62.95
C-Peptide	$CP_{2b}$	Peripheral C-peptide concentration	pmol/L	987.25

**Figure A1:**
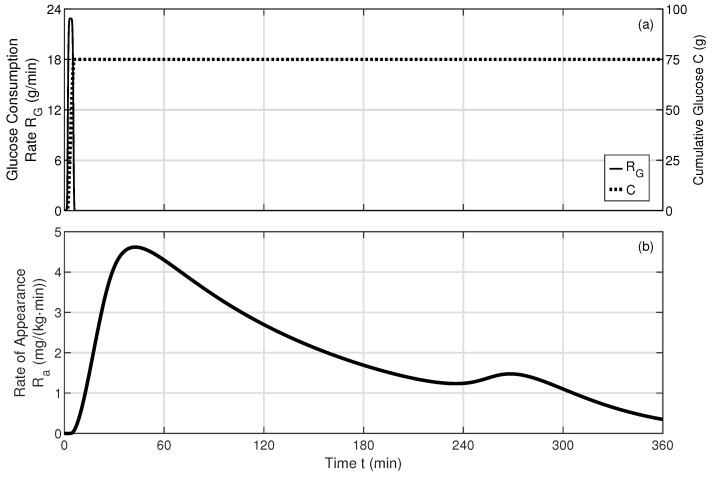
Differential-equation approach to modeling the glucose consumption rate ($R_{G}=D\cdot d\left(t\right)$) appearing in Equation (D3) of Figure 1. (**a**) For the study shown in the bottom row of Figure 4, $R_{G}$ emulates OGTT conditions; the cumulative consumed glucose (*C*) rises to 75 g within five minutes. (**b**) Rate of appearance ($R_{a}$) found by including $R_{G}$ in the system of derivative functions used by the ODE solver.

## Data Availability

The original contributions presented in this study are included in the article. Further inquiries can be directed to the corresponding author.

## Abbreviations

The following abbreviations are used in this manuscript:

ADA	American Diabetes Association
DM	Diabetes mellitus
JSPS	Japanese Society for the Promotion of Science
LADA	Latent autoimmune diabetes of adults
MODY	Maturity-onset diabetes of the young
ODE	Ordinary differential equation
OGTT	Oral glucose tolerance test
T1DM	Type 1 diabetes mellitus
T2DM	Type 2 diabetes mellitus

## Appendix A. Physiological Model Parameters

Model parameters used throughout the manuscript are reported in Table A1, which lists the parameter values for median early-stage T2DM, as well as their sources, physiological subsystems, and inclusion in the basal model. The $r_{1}$ and $r_{2}$ risk parameters are set to zero because the risk function (risk), whose value they help govern, equals zero when $G\geq G_{b}$, as defined in Equation (D16); all basal and non-erroneouspostprandial *G* values investigated in this work abide by this condition.

**Table A1:** Physiological model parameters organized by subsystem and flagged (using purple text and with “Yes”) as to their presence in the basal-state model. Values represent the median values of non-insulin-dependent, early-stage T2DM. The glucose, insulin, and C-peptide systems are separated by double lines.

Subsystem	Symbol	Description	Units	Value	Origin	Basal?
Rate of Appearance	$k_{max}$	Maximum gastric emptying rate	1/min	0.0426	[18]	No
	$k_{min}$	Minimum gastric emptying rate	1/min	0.0076	[18]	No
	$k_{abs}$	Intestinal absorption rate	1/min	0.0542	[18]	No
	$k_{gri}$	Stomach-grinding rate	1/min	0.0465	[14]	No
	*f*	Intestinal absorption fraction	Unitless	0.9	[18]	No
	*b*	Gastric-emptying reduction inflection point	Unitless	0.73	[18]	No
	*c*	Gastric-emptying recovery inflection point	Unitless	0.1	[18]	No
	$\psi_{b}$	Gastric-emptying reduction mass rate	1/mg	0.00010	(D2)	No
	$\psi_{c}$	Gastric-emptying recovery mass rate	1/mg	0.00037	(D3)	No
Glucose Kinetics	$k_{1}$	Rate parameter	1/min	0.066	[18]	Yes
	$k_{2}$	Rate parameter	1/min	0.043	[18]	Yes
	$V_{G}$	Glucose distribution volume	dL/kg	1.00	[18]	Yes
Glucose Excretion	$k_{e1}$	Glomerular filtration rate	1/min	0.0005	[18]	Yes
	$k_{e2}$	Glucose excretion threshold	mg/kg	339	[18]	Yes
	*s*	Excretion switch parameter	Unitless	0 or 1	(5b)	Yes
Endogenous Glucose Production	$k_{p1}$	Extrapolated EGP for zero glucose and insulin	mg/(kg·min)	3.09	[14]	Yes
	$k_{p2}$	Hepatic glucose effectiveness	1/min	0.0008	[18]	Yes
	$k_{p3}$	Hepatic insulin sensitivity	$\frac{\mathrm{mg}/(\mathrm{kg}\cdot \min)}{\mathrm{pmol}/L}$	0.0060	[18]	Yes
	$k_{p4}$	Portal insulin sensitivity	$\frac{\mathrm{mg}/(\mathrm{kg}\cdot \min)}{\mathrm{pmol}/\mathrm{kg}}$	0.0484	[18]	Yes
Glucose Utilization	$F_{cns}$	Insulin-independent glucose utilization	mg/(kg·min)	1	[18]	Yes
	$V_{m0}$	Michaelis–Menten parameter	mg/(kg·min)	4.65	[14]	Yes
	$K_{m0}$	Michaelis–Menten parameter	mg/kg	466.2	[18]	Yes
	$V_{mx}$	Insulin sensitivity to glucose utilization	$\frac{\mathrm{mg}/(\mathrm{kg}\cdot \min)}{\mathrm{pmol}/L}$	0.034	[18]	No
	$r_{1}$	Risk function parameter	Unitless	0	Appendix A	No
	$r_{2}$	Risk function parameter	Unitless	0	Appendix A	No
	$G_{th}$	Hypoglycemic threshold	mg/dL	60	[17]	No
Insulin Secretion	α	Delay between glucose and insulin secretion	1/min	0.034	[18]	No
	$\Phi_{s}$	Beta-cell responsivity to glucose	$10^{-9}$/min	20.30	[18]	No
	$\Phi_{d}$	Beta-cell responsivity to glucose rate of change	$10^{-9}$	286.0	[18]	No
	*h*	Glucose threshold for beta-cell secretion	mg/dL	$G_{b}$	(D20)	No
Insulin Kinetics	$m_{1}$	Rate parameter	1/min	0.314	[18]	Yes
	$m_{2}$	Rate parameter	1/min	0.268	[18]	Yes
	$m_{4}$	Rate parameter	1/min	0.443	[18]	Yes
	$m_{5}$	Rate parameter	1/min	0.260	[18]	Yes
	$m_{6}$	Rate parameter	1/min	0.017	[18]	Yes
	$\alpha_{G}$	Glucose control on HE	dL/mg	0.005	[18]	Yes
	$\alpha_{0G}$	Extrapolated HE for zero glucose	Unitless	1.16	[14] †	Yes
	$V_{I}$	Insulin distribution volume	L/kg	0.041	[18]	Yes
	$k_{i}$	Insulin-action delay rate	1/min	0.0075	[18]	No
	$p_{2u}$	Rate of insulin action on glucose utilization	1/min	0.058	[18]	No
C-Peptide Kinetics	$k_{01}$	Rate parameter	1/min	0.062	[23]	Yes
	$k_{12}$	Rate parameter	1/min	0.051	[23]	Yes
	$k_{21}$	Rate parameter	1/min	0.053	[23]	Yes
	$V_{C}$	C-peptide distribution volume	L	4.18	[23]	Yes
	$CP_{1b}$	Basal plasma C-peptide concentration	pmol/L	950	[18] †	Yes

Origin-column notation. [ ]: explicit value provided in a reference. [ ] †: Estimated value based on a reference. ( ): Calculated value based on an equation. A: Value chosen by the authors, as discussed in the text of Appendix A.

## Appendix B. Dynamic Model Solver and Oral Glucose Consumption

The dynamic model is solved in this work using an ordinary differential equation (ODE) solver in MATLAB. The ode15s solver is used due to its general appropriateness for both stiff and non-stiff problems [28], although several other solvers yield matching results. The differential equations in Figure 1 are used to build the system of derivative functions required for the ODE solver.

Additionally, we expand this system of derivative functions to include oral glucose, appearing in Equation (D3) as D·d(t), where *D* is the total consumed glucose and d(t) is its rate of distribution over time. Specifically, its derivative function is expressed as

(A1) $$\frac{dC(t)}{dt}=R_{G}\left(t\right)=D\cdot d\left(t\right),$$

where *C* is the cumulative consumed glucose and $R_{G}$ is the oral glucose consumption rate. This formalism allows d(t) to take on any shape, and we model it here as a super Gaussian function:

(A2a) $$\begin{array}{cc} d(t) & =\frac{1}{N}\exp\left(-{\left(\frac{{\left(t-t_{o}\right)}^{2}}{2\sigma^{2}}\right)}^{m}\right), \end{array}$$

(A2b) $$\begin{array}{cc} N & =\int_{t_{start}}^{t_{end}}\exp\left(-{\left(\frac{{\left(t-t_{o}\right)}^{2}}{2\sigma^{2}}\right)}^{m}\right)\;dt, \end{array}$$

where $t_{0}$ is the time of peak glucose consumption; σ is the standard deviation; *m* is the order of the super Gaussian function; $t_{start}$ and $t_{end}$ are the times at the beginning and end of the ODE-solver run, respectively; and the normalization term (*N*) forces ∫ddt=1. The ODE solver integrates $R_{G}$ over time and yields C(t).

For the dynamic study shown in the bottom row of Figure 4, parameter values are chosen to emulate the requirements of the OGTT [25]. Setting m=3, σ=1.25 min, $t_{0}=3\sigma =3.75$ min, $t_{start}=0$ min, and $t_{end}=360$ min yields a relatively flat-top *d* profile defined by a third-order super Gaussian function centered at t=3.75 min with a full-width at half-maximum value of 3.5 min, as shown in Figure A1a. The consumed glucose (*C*) rises over this time span and settles at D=75 g. The resulting rate-of-appearance (Ra) profile is shown in Figure A1b.
